# Autophagy: roles in intestinal mucosal homeostasis and inflammation

**DOI:** 10.1186/s12929-019-0512-2

**Published:** 2019-02-14

**Authors:** Sabah Haq, Jensine Grondin, Suhrid Banskota, Waliul I. Khan

**Affiliations:** 1 Farncombe Family Digestive Health Research Institute, McMaster University, Hamilton, ON L8N 3Z5 Canada; 2 Department of Pathology and Molecular Medicine, McMaster University, Room 3N7, Hamilton, ON L8N 3Z5 Canada

**Keywords:** Autophagy, Intestinal mucosa, Epithelium, Immune response, Inflammation, Microbiota

## Abstract

The intestinal mucosa is a site of multiple stressors and forms the barrier between the internal and external environment. In the intestine, a complex interplay between the microbiota, epithelial barrier and the local immune system maintains homeostasis and promotes a healthy gut. One of the major cellular catabolic processes that regulate this homeostasis is autophagy. Autophagy is required to maintain anti-microbial defense, epithelial barrier integrity and mucosal immune response. Dysregulation of the autophagy process causes disruption of several aspects of the intestinal epithelium and the immune system that can lead to an inappropriate immune response and subsequent inflammation. Genome-wide association studies have found an association between several risk loci in autophagy genes and inflammatory bowel disease. The aim of the current review is to provide an update on the role of autophagy in intestinal mucosal physiology and in the control of inappropriate inflammation.

## Introduction

Autophagy or “self-eating” is the catabolic process of delivering cytoplasmic constituents, organelles and infectious agents to the lysosome for degradation [1]. It is a highly conserved mechanism that takes place in all eukaryotic cells and participates in maintaining normal physiology [1]. During starvation, growth factor deficiency or high-energy demand, autophagy is induced to generate energy that supports metabolic processes [1]. Apart from starvation, autophagy is critical in responding to a diverse range of stressors namely, hypoxia, infection, endoplasmic reticulum (ER) stress, tissue remodeling, cellular debris breakdown, turnover of damaged organelles, tumor suppression, immune response, and cell death [2, 3]. Basal autophagy i.e. the baseline level of autophagy maintained as a housekeeping function in all cells, contributes to routine cell turnover and is important for the maintenance of cellular homeostasis [1, 4]. Autophagy results in the clearance of polyubiquitinated protein aggregates, which are formed during stress, aging, and disease. Autophagy also degrades invading pathogens, modulates the release of pathogen induced pro-inflammatory cytokines and participates in antigen presentation and lymphocyte development [2, 3]. Furthermore, this process is essential in maintaining intestinal barrier integrity, anti-microbial defense and mucosal immune response [5]. Even though autophagy is a survival mechanism, it can lead to cell death either in association with apoptosis or as an alternative mechanism [6]. The various roles of autophagy in regulating homeostasis and inflammation are extremely significant in the context of the intestinal mucosa, where most of the stressors are likely to converge [5]. The intestinal mucosa is constantly exposed to the gut microbiota and food particles since it is adjacent to the gut lumen. The epithelial and the resident immune cells of the mucosa are essential for defense against these antigens. Dysregulation of autophagy has been implicated in the pathogenesis of various diseases, including inflammatory bowel disease (IBD) [7]. The two major forms of IBD, Crohn’s disease (CD) and ulcerative colitis (UC), are serious chronic inflammatory conditions of the human bowel [7]. In spite of decades of research, IBD pathophysiology is not fully understood. The development and course of IBD are affected by several factors, including genetic susceptibility of the host, the intestinal microbiota, other environmental factors, and the host immune system [8]. Genome-wide association studies (GWAS) revealed that polymorphisms in autophagy genes contribute to the development of IBD [9]. Therefore, understanding the role of autophagy in intestinal homeostasis and pathogenesis of inflammation is important to the development of new strategies in prevention and/or treatment of intestinal inflammatory diseases.

The purpose of the review is to provide the readers with an update on the mechanisms of autophagy and present understanding on the role of autophagy in intestinal homeostasis and inflammation. The role this process plays during the inflammatory response in both patients and in experimental models will also be examined. In addition, the emerging relationship between gut microbiota and autophagy has also been discussed due to its significance in the context of gut inflammation and possible future therapies.

### Mechanisms of autophagy

Autophagy comprises three major intracellular pathways in eukaryotic cells: macroautophagy, microautopahgy, and chaperone-mediated autophagy (CMA). These pathways share a common destiny of lysosomal degradation but differ mechanistically [1, 2]. In macroautophagy, double membrane-bound vesicles called autophagosomes are formed that engulf cytoplasmic constituents and organelles. The autophagosomes fuse with lysosomes, where the contents are degraded. Microautophagy, by contrast, involves direct engulfment and degradation of the target components in the lysosome. Microautophagy is important in the maintenance of organelles and membranes, as well as, cell survival during starvation. However, it is not clear whether it occurs simultaneously with macroautophagy or if it is simply a compensatory mechanism for macroautophagy to clear the excess metabolic materials [10]. In CMA, cytosolic chaperone heat shock cognate 70 kDa protein (HSC70) binds to a KFERQ-like pentapeptide motif of the target protein. HSC70 then associates with lysosome-associated membrane protein 2A (LAMP2A) resulting in its oligomerization. This promotes the translocation of the targets across lysosomal membranes into the lysosomal lumen [2]. Among the three autophagy pathways, macroautophagy has been extensively studied and linked to many intestinal physiological and pathological processes. In this review, we will focus on macroautophagy and, from this point on, this process will simply be referred to as autophagy.

**Fig. 1:**
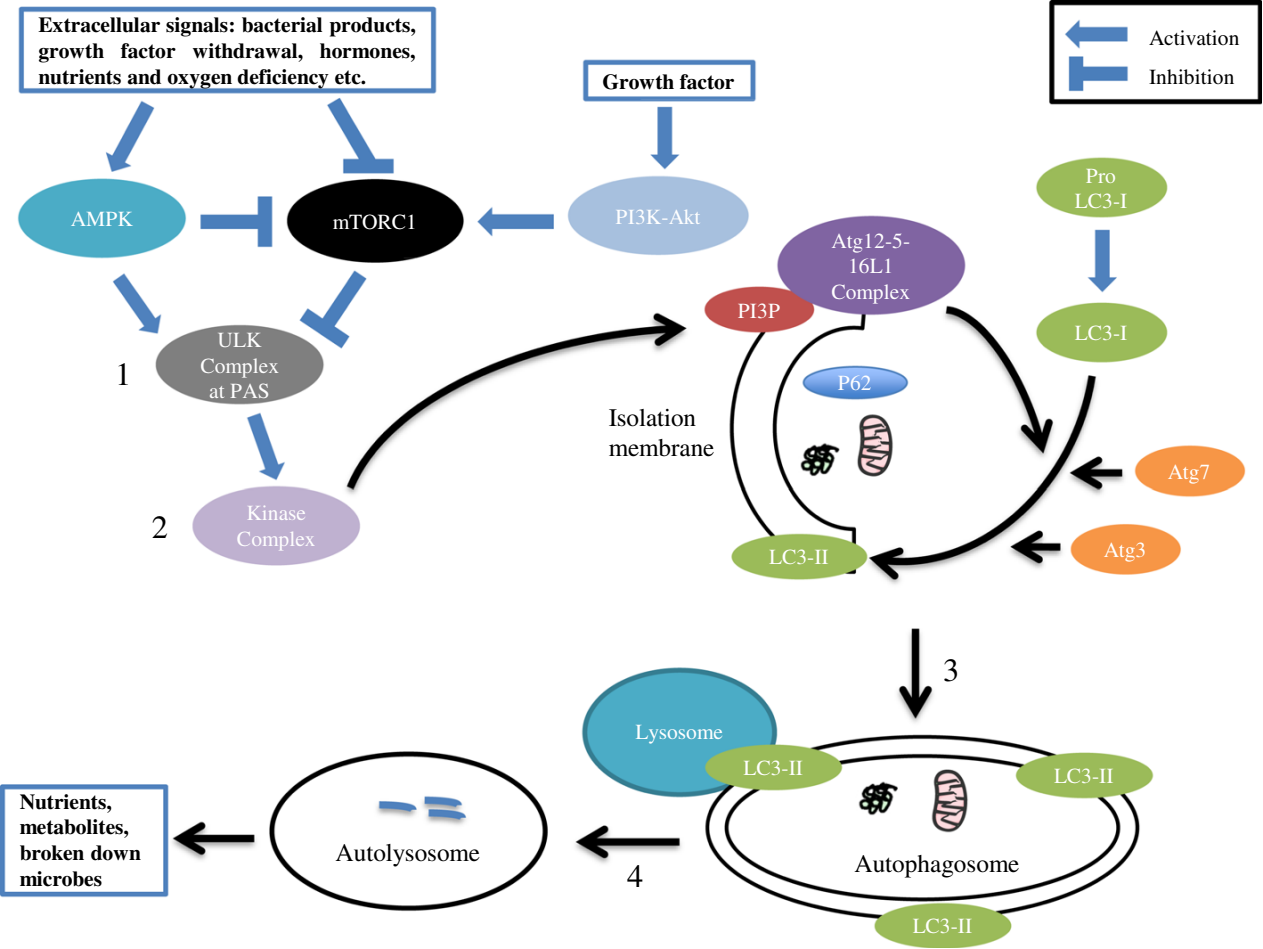
Mechanisms of macroautophagy. Stress signals activate AMPK and inhibits mTORC1. mTORC1 is inhibited by AMPK and activated by growth factor induced PI3K-Akt pathway that initiates autophagy. 1. Initiation: Activated AMPK and inhibited mTORC1 initiates ULK complex. 2. Nucleation: ULK complex recruits kinase complex to the PAS that results in the production of PI3P at the isolation membrane. 3. Elongation: PI3P recruits downstream Atg proteins for elongation of the membrane. Atg12–5 forms a complex with Atg16L1 that associates with isolation membrane. The Atg12–5-16 L1 complex along with Atg7 and Atg3 are required for the formation of LC3-II from LC3-I. The LC3-II mediates the elongation and completion of the autophagosome. 4. Fusion: Autophagosome fuses with the lysosome to form the autolysosome. Lysosomal enzymes degrade the contents of the autolysosome. In selective autophagy, adaptor proteins like p62 binds to ubiquitinated proteins and organelles, attaches them with LC3-II and brings them in the autophagosome for degradation

The mechanisms of autophagy have been studied originally in the yeast model. Since autophagy is highly conserved, the pathways found in yeast can be translated to mammalian cells [5]. The mechanism has been summarized in Fig. 1. The central upstream regulators of autophagy are mammalian target of rapamycin complex 1 (mTORC1) and adenosine monophosphate-activated protein kinase (AMPK). AMPK activates the process of autophagy where as mTORC1 inhibits it. Nutrient sufficiency and growth factor stimulation activates mTORC1 resulting in inhibition of autophagy [11, 12]. On the other hand, starvation, growth factor withdrawal and ER stress activate AMPK that, in turn, inhibits mTORC1 and activates autophagy [11, 12]. The opposing roles of mTORC1 and AMPK in the autophagy pathway are due to phosphorylation of Unc-51 like autophagy activating kinase (ULK1) at two different sites [13]. Some of the other upstream regulators of autophagy pathway are class I phosphatidylinositol-3-kinase (PI3K), p53, death associated protein kinase and hypoxia inducible factor 1 (HIF-1) [14].

One of the hallmarks of autophagy is the formation of autophagosome, a double membrane bound vesicle containing portions of cytoplasm. Autophagosome formation occurs through initiation, nucleation and elongation of the isolation membrane in the phagophore assembly site (PAS). The origin of the isolation membrane is unclear and is thought to be derived from either the ER, the Golgi or the plasma membrane. The ULK complex composed of ULK1, autophagy-related protein 13 (Atg13), focal adhesion kinase family interacting protein of 200 kDa (FIP200) and autophagy-related protein 101 (Atg101), initiates autophagosome formation in the PAS. In the nucleation stage, the ULK1 complex recruits beclin-1, autophagy-related protein 14 (Atg14), vacuolar protein sorting 15 (Vps15) and class III phosphatidylinositol-3-kinase (PI3K) to the PAS [15]. Class III PI3K converts phosphatidylinositol to phosphatidylinositol-3-phosphate (PI3P) at the PAS. PI3P forms a platform for the binding of the downstream autophagy proteins to the isolation membrane of the forming autophagosome and allows this membrane to be deformed, bent and expanded. During the elongation stage autophagy-related protein 12–5 (Atg12–5) conjugate is activated by the enzymes autophagy-related protein 7 (Atg7) and autophagy-related protein 10 (Atg10). Atg12–5 forms a complex with autophagy-related protein 16L1 (Atg16L1) that associates with isolation membrane and functions as an ubiquitin-like enzyme. The Atg12–5-16L1 complex is required for the lipidation of the microtubule-associated protein 1 light chain 3 (LC3-I) with phosphatidylethanolamine (PE) to form LC3-II [16]. The lipidation of LC3-I to LC3-II is catalyzed by the enzymes Atg7 and Atg3 [17]. LC3-II is hydrophobic which allows for easier incorporation into, elongation of the isolation membrane, and closure of the autophagosome [17]. Selective autophagy is an autophagic process where cytoplasmic components are selected, tagged and delivered to the forming autophagosome, where as non-selective autophagy focuses on the bulk degradation of cytoplasmic components [18]. In selective autophagy, adaptor protein like p62 binds to ubiquitinated proteins and organelles, attaches them with LC3-II that helps bring them into autophagosome [17, 18]. In the final stage of its formation, the autophagosome fuses with the lysosome to form autolysosome, a process mediated by proteins like Rab7, syntaxin 17, vesicle-associated membrane protein 8 (VAMP8) and LAMP2. The inner membrane and contents of the autolysosome are broken down by enzymes such as hydrolases and released into the cytoplasm, thus completing the process of autophagy [1, 14]. This brief overview of mammalian autophagy pathways demonstrates the complexity of the process (Fig. 1). It should be mentioned that, recent discoveries of alternative autophagic pathways such as the Atg5/Atg7 independent autophagy pathway, suggest that different autophagy pathways and autophagy-related molecules may exist in different types of cells [19]. These differences may be due to variances in stressors or in functions of specific cell types. For example, in the intestine the mechanism and molecules associated with the process of autophagy may be altered in specialized secretory epithelial cells such as Paneth, goblet and enteroendocrine (EE) cells in comparison with absorptive epithelial cells. However, further investigation is needed to elucidate this notion.

### Autophagy in intestinal epithelium

The intestinal epithelium, the barrier between the lumen and intestinal mucosa, is constantly exposed to food and microbes and acts as the first line of defense against microbial invasion [5, 20]. Intestinal epithelial cells (IECs), as well as, the specialized epithelial cells such as enteroendocrine (EE) cells, goblet cells, Paneth cells, microfold (M) cells and tuft cells make up this single layer that acts as the site of digestion and absorption [3]. Basal autophagy in M cells is relatively low compared to other epithelial cells since the function of M cells is simply to transport antigens from the gut lumen through the epithelial layer to the underlying Peyer’s patches. However, when mice are exposed to stresses such as cigarette smoke, autophagy is induced in both M cells and follicle associated epithelium. Even though, in the Peyer’s patches of the murine ileum, autophagy levels are increased in response to cigarette smoke exposure, some of the downstream autophagy proteins does not show a significant increase compared to the air exposed control mice. It is thought that smoking results in oxidative stress that induces autophagy to repair the cell damage [21]. It can be assumed that the findings in B and T cells might be applicable to the PP since PP is in fact an aggregate of lymphoid follicles with germinal B cell center surrounded by T cell zone. Since the relationship between autophagy and M cells and tuft cells has not been explored sufficiently, future research needs to be conducted in this area. In the intestine, a sensitive balance between tolerance and defense is required to maintain homeostasis. The conserved process of autophagy plays a critical role in maintaining this balance by regulating the functions of these different types of cells in the mucosal layer [5].

### Intestinal epithelial cells

Epithelial cells are the first line of defense in host tissues against various invading gut microbiota and pathogens [22, 23]. In IECs, autophagy is activated in response to invasive bacteria, such as *Salmonella enterica*, *Enterococcus faecalis* and *Shigella,* in order to inhibit their replication, invasion and dissemination. The activation of autophagy is indicated by the presence of autophagosomes in the epithelial cells marked by LC3 puncta [23–25]. The interaction between autophagy and the toll-like receptors (TLRs) signaling pathway has also been studied in the IECs in vitro. It has been shown that IECs have high levels of autophagy that is not upregulated upon stimulation of TLR-2 or 4 or 5. However, when basal autophagy is silenced by Atg7 siRNA transfection in the IEC lines, there is decreased TLR-2, or 4 or 5 mediated interleukin 8 (IL-8) production [26]. On the contrary, Fujishima and his group have shown up-regulation of expressions of interleukin 1β (IL-1β) and tumor necrosis factor α (TNF-α) mRNA by lipopolysaccharide (LPS) in *Atg7* deficient murine small intestinal epithelium compared to control epithelium [27]. These contrasting findings between in vitro and in vivo systems suggest for taking caution in extrapolation of in vitro findings in relation to biological system and warrants further studies on the role of autophagy in intestinal epithelium. Furthermore, intestinal epithelial barrier integrity is regulated by autophagy [28–30]. Autophagy increases tight junction barrier function in Caco-2 IECs by enhancing the lysosomal breakdown of pore forming tight junction protein claudin-2 [28]. Similarly, autophagy activation in porcine IECs by rapamycin demonstrates a partial rescue of non-essential amino acid deprivation induced barrier dysfunction [29]. On the contrary, rapamycin mediated induction of autophagy in Caco-2 IECs has opposite effects on intestinal barrier function. Increased autophagy causes reduced transepithelial electrical resistance, enhanced paracellular permeability and disruption of zonula occludens-1 and occludin [30].

### Paneth cells

Among the specialized cells of epithelial layer, the importance of autophagy in Paneth cell function has been extensively studied and has been emphasized in the context of CD. Paneth cells, located in the crypts of Lieberkuhn of the small intestine, store and secrete anti-microbial peptides (AMPs), such as lysozyme, α-defensin and phospholipase A2. The AMPs contribute to the maintenance of healthy gut microbiota [31]. Autophagy is an important regulator of Paneth cell function. *Atg16L1* and *Atg5* hypomorphic mice have abnormal granule exocytosis in Paneth cells that interferes with the secretion of AMPs and bacterial killing [32]. Autophagy gene deficient Paneth cells also show increased expression of genes involved in peroxisome proliferator-activated receptor signaling and production of leptin and adiponectin, both of which are involved in intestinal injury response. Similar changes in Paneth cells in CD patients carrying the *Atg16L1* risk allele have been reported [32]. Different groups have provided similar evidence of reduced granule size and decreased lysozyme staining in Paneth cells in IEC specific *Atg7* conditional knock out (KO) mice [33], in *Atg4B* null mice [34] and in *Atg16L1 T300A* knock in mice [35]. The secretion of lysozyme from Paneth cells during bacterial infection takes place through an autophagy-based alternative secretion pathway triggered by bacteria-induced ER stress [36]. However, a study found that mouse enteral starvation induces autophagy in Paneth cells, decreases AMP production and increases translocation of bacteria to mesenteric lymph nodes [37]. These contradictory findings may be explained as an attempt of the Paneth cells to maintain vital functions at the expense of the physiological function of autophagy. During starvation, induced autophagy results in the synthesis of new constituents. These new constituents are used for production of proteins essential for cell survival instead of proteins such as AMPs. Other factors such as a change in the microbial composition during starvation could also influence Paneth cell AMP activity [37]. The aforementioned evidence emphasizes the importance of autophagy in regulation of Paneth cell function and AMP generation, packaging and secretion [38].

### Goblet cells

Goblet cells are specialized cells that are responsible for the production and preservation of the protective mucus blanket by producing high molecular weight glycoproteins known as mucins. This mucus layer is an important component of the intestinal anti-microbial systems that effectively separate the gut microbiota and intestinal epithelium and help maintain homeostasis. Up to 21 different mucin genes have been identified in humans, and the majority of their homologues have been recognized in mice and rats [39]. Among these mucin genes, MUC2 (Muc2 in mice) is the major gel forming mucin in the gut, which is the most important factor determining goblet cell morphology [40]. *Muc2* deficient mice develop spontaneous colitis and are more susceptible to dextran sulphate sodium (DSS) mediated model of colitis [41]. Additionally, in models of enteric infections, Muc2-dependent mucus production has been shown to be critical in host protection. By utilizing *Muc2* deficient mice and mice that are resistant (C57Bl/6) or susceptible (AKR) to *Trichuris muris* infection, studies have illustrated Muc2 mucin is an important component of innate defense in this model of parasite infection [42]. An important role of Muc2 in host defense is also shown in *Citrobacter rodentium* infection (mouse model of enteropathogenic *E.Coli* (EPEC) and enterohemorrhagic *E.Coli* (EHEC)) [43]. Autophagy plays an important role in goblet cell functions. Through the production of reactive oxygen species, autophagy protein LC3 regulates the accumulation and secretion of mucin granules from colonic goblet cells. Reactive oxygen species are produced in part by LC3 positive vacuole associated nicotinamide adenine dinucleotide phosphate hydrogen (NADPH) oxidase. Thus, autophagy and NADPH oxidase activity drive reactive oxygen species production that is critical for efficient mucin formation and secretion by goblet cells [44]. In addition, mice with defective autophagy are reported to have enlarged goblet cells in the intestinal epithelial layer [35]. Wlodarska and his group [45] further reinforced the role of autophagy in the control of goblet cell function. They showed that nucleotide-binding oligomerization domain protein-like receptors protein 6 (NLRP6) inflammasome regulates goblet cell mucus secretion through the induction of autophagy [45]. *NLRP6* deficient mice exhibit defective autophagy in goblet cells and reduced mucus secretion in the large intestinal lumen. LC3-GFP tagged *NLRP6* deficient mice have no LC3-GFP signal, reduced amounts of LC3-GFP protein and accumulation of p62 in the intestinal epithelium, indicating defective autophagy. In addition, increased LC3-I/II ratio and degenerating mitochondria are also observed in the intestinal epithelium of *NLRP6* deficient mice. In order to further establish a link between impaired autophagy and goblet cell function, *Atg5*^*+/−*^ mice were used to study the effects on goblet cells. Even a partial loss of autophagy function leads to goblet cell hyperplasia, perturbations in the secretory pathway and defects of the mucus layer. It was concluded from these results that inflammasome signaling is critical in maintaining a healthy goblet cell secretory function via autophagy. Further investigations in both Paneth and goblet cells is required to determine whether other mechanisms, apart from ER stress and reactive oxygen species production, are involved in autophagy-induced secretion.

### Enteroendocrine cells

With enteroendocrine (EE) cells producing more than 30 different hormones, the gut is the largest endocrine organ in the body [46]. The enteric hormones such as, serotonin (5-hydroxytryptamine; 5-HT), chromogranin, cholecystokinin, secretin, glucagon-like peptide 1 and 2 (GLP-1, GLP-2), produced by EE cells are involved in a range of physiological and pathological functions [46]. Change in EE cell number and secretion patterns have been documented in patients with intestinal inflammation. There are reports of increased number of peptide YY and chromogranin A expressing cell (CgA+) density in lymphocytic colitis, as well as, increased GLP-1, GLP-2 secreting cells and increased 5-HT secreting enterochromaffin (EC) cells in IBD [46–48]. Recent evidence shows that EE cells are regulated by autophagy. CgA+ EE cells are increased in the colon of IBD patients and as well as in DSS induced mouse model of colitis. The number of CgA+ EE cells is also increased in the colonic mucosa of mice following treatment with pro-inflammatory cytokines interferon γ (IFN-γ) and TNF-α. In addition, increased levels of autophagy markers, LC3-II and Atg5, are detected in the colonic mucosa of these cytokine treated mice. In IFN-γ and TNF-α treated mice, the number of CgA+ cells are not increased following treatment with the autolysosome inhibitor, chloroquine. Furthermore, it has been shown that the increase in the number of CgA+ cells in the mucosa of DSS colitic mice is prevented following administration of chloroquine. These findings show an important role of autophagy in CgA production and differentiation of CgA+ cells during inflammation [49]. Recently, *Drosophila* guts lacking WD-40 domain of *Atg16* was found to have reduced number of mature EE cells, due to a dysfunctional Robo-Slit signaling pathway. Pre-EE cells of the *Atg16* gut mutant *Drosophila* model failed to mature into fully differentiated EE cells. The pre-EE cells containing mutant *Atg16* failed to produce enough slit (a regulator protein of EE cell fate) as evidenced by reduced mRNA and protein levels. This may have resulted in accumulation of pre-EE cells in the gut. The *Atg16 Drosophila* gut mutant also showed elevated levels of pro-inflammatory cytokines and increased susceptibility to DSS colitis. Moreover, it is evident that *Drosophila Atg16* promotes EE cell differentiation from intestinal stem cells [50]. The above stated evidence indicates a link between EE cells, autophagy and colitis. The relationship between EE hormones and intestinal inflammation has been extensively studied in experimental models of colitis. Mice are less susceptible to DSS and dinitrobenzenesulphonic acid (DNBS) induced colitis when gut 5-HT content is reduced either by using Tph enzyme inhibitor or by knocking out of the *Tph1* gene [51]. The relationship between 5-HT and gut inflammation is further supported by reports of exaggeration in trinitrobenzesulphonic acid (TNBS) colitis in serotonin reuptake transporter (*SERT*) KO mice [52]. Recently, some EE hormones have been investigated as possible regulators of autophagy. There is evidence of autophagy inhibition by 5-HT in hepatocellular carcinoma cells, in the lacrimal gland and in the rat hippocampus [53–55]. GLP-2, another EE hormone has also been shown to robustly activate the mTORC1 pathway in murine small intestine [56]. Exogenous parenteral administration of GLP-2 in C57BL/6 mice increases the phosphorylation of eukaryotic translation initiation factor 4E (eIF4e)-binding protein 1 (4E-BP1) and S6 ribosomal protein, both of which are downstream effector proteins of the mTORC1 pathway. Pretreatment of the mice with rapamycin, an mTOR inhibitor, before administration of GLP-2 reduces levels of phosphorylated 4E-BP1 and S6 ribosomal protein. This shows that GLP-2 activates the mTORC1 pathway, which regulates autophagy. These findings suggest presence of interactions between EE hormones and autophagy process in both intestinal and extra intestinal sites. Among the various cell types in the intestinal mucosa, the role of autophagy in EE cells is underexplored within the context of intestinal inflammation. While dysregulated EE cell signaling and autophagy have been implicated in intestinal inflammation, it remains unclear whether they interact with each other in relation to intestinal pathology and pathophysiology, particularly in inflammation. Due to the vital roles of the gut hormones in the maintenance of intestinal homeostasis, it is of utmost importance to further study the relationship between EE hormones and the autophagy system.

### Autophagy in intestinal immune cells

The intestinal mucosal immune system directs the appropriate immune response to a vast array of microbial and dietary challenges. The gut is one of the largest reservoirs of resident immune cells [38]. Macrophages, dendritic cells (DCs), T cells, B cells, and natural killer cells are important components of the intestinal mucosal immune system. Recent compelling evidence has revealed autophagy as a key regulator of intestinal innate and adaptive immunity [57].

### Macrophages

Autophagy regulates the function of pathogen clearance in macrophages. For example, Atg7 and Atg5 expression in macrophages is essential for clearance of *Pseudomonas aeruginosa* and *Toxoplasma gondii* [58, 59]. In macrophages, TLR signaling links the components of the classical autophagy pathway and phagocytosis. The autophagy proteins LC3 and beclin-1 are rapidly recruited to the phagosome following ligand binding to TLR-2 on murine macrophages. LC3 and beclin-1 are associated with rapid fusion of the phagosome and the lysosome, leading to enhanced killing of ingested organisms like *Mycobacterium tuberculosis* [60]. However, the response of *Atg16L1* deficient mice to *C. rodentium* infection is very different compared to that of other pathogens [61]. *Atg16L1* deficient mice are resistant to *C. rodentium* infection due to a monocyte mediated heightened immune response. This illustrates an unexpected role of Atg16L1 in reducing a beneficial immune response to enteric bacterial infection. Recently, evidence showed that gut microbiota mediated type I IFN (IFN-1) signaling pathway maybe responsible for the heightened immune response [62]. The process of autophagy counteracts a spontaneous IFN-I response to gut microbiota that is beneficial in the presence of infectious and non-infectious intestinal hazards and injury. In autophagy deficient mice, the gut microbiota contributes to spontaneous IFN-I signaling resulting in enhanced resistance to *C. rodentium* infection. Conversely, inflammatory cytokines also influence autophagy. It has been reported that the Th1 type cytokines, such as IFN-γ, produced during bacterial infection activates autophagy as opposed to the inhibitory Th2 response [57]. Autophagy has crucial roles in the transcription, degradation and secretion of pro-inflammatory cytokines highlighting its importance in cytokine signaling. Macrophages from *Atg16L1* deficient mice have been documented to produce increased amounts of LPS induced IL-1β, IL-18 and TNF-α [63–65]. Fetal liver cell chimeric mice with *Atg16L1* deficient hematopoietic cells have increased susceptibility to DSS colitis as demonstrated by acute weight loss and severe distal colon inflammation suggesting a protective role of autophagy in the suppression of colitis [63]. In addition, these chimeric mice have increased levels of pro-inflammatory cytokines IL-1β and IL-18 in the sera after DSS treatment. Treatment with neutralizing antibodies for these cytokines reduce the susceptibility of *Atg16L1* deficient mice to DSS induced colitis, indicating the importance of Atg16L1 in the suppression of intestinal inflammation [63]. Cd11b + DCs and macrophages isolated from *Atg16L1 T300A* knock in mice also express increased levels of LPS induced IL-1β and decreased anti-bacterial autophagy [35]. Similarly, human macrophages isolated from patients with CD *Atg16L1 T300A* variant are reported to produce higher levels of interferon β (IFN-β) and IL-1β [66]. Both *Atg16L1* deficient chimeric and *Atg16L1 T300A* knock in mice are autophagy deficient.

### Dendritic cells

Dendritic cells are one of the key components of the immune system that establish the link between innate and adaptive immunity. DCs initiate immune response by antigen uptake, presentation and activation of T cells. As demonstrated by different research groups, autophagy regulates interactions between DCs and T cells [67–69]. Using DC-IECs co-culture system, Strisciuglio and his group [67] showed that DCs form decreased transepithelial protrusions when autophagy is reduced in either DCs or epithelial cells. This leads to reduced antigen sampling and IL-10 secretion, increased DC maturation, increased T cell proliferation and production of pro-inflammatory type of DC [67]. Cooney and his group reported a decrease in bacterial trafficking, antigen presentation and T cell response to pathogens when *Atg16L1 T300A* allele is expressed in DCs [68]. Similarly, CD4^+^ T cell activation is reduced in mice with *Atg5* deficient DCs. This is due to defective processing of cytosolic, phagocytosed and soluble antigens for major histocompatibility complex (MHC) II loading in DCs [69]. Other antigen presenting cells (APC) such as B cells also depend on intact autophagy machinery for endogenous antigen presentation [70]. Intracellular microbes such as *M. tuberculosis* and *Listeria monocytogenes* are degraded into small peptides in the autophagolysosomes and loaded on the MHC II of APC to activate CD4^+^ T cells [3].

### T and B cells

Autophagy regulates activation and maintenance of T cells. In mice, T cell specific deletion of autophagy genes *Atg3, Atg5, Atg7, Atg16L1* or *beclin-1* results in decreased numbers of CD4^+^, CD8^+^ and Treg cells, defective effector and memory T cell development, and expansion of intestinal Th2 cells [71–73]. Kabat et al. [74] reported a reduction in the survival of intestinal Foxp3^+^ regulatory T (Treg) cells when *Atg16L1* gene is deleted, which leads to severe gut inflammation in the mice [74]. Additionally, autophagy protein, Vps34, regulates the development, T cell receptor (TCR)-induced proliferation and apoptosis of T cells in the thymus and periphery. *Vps34* deficient T cells shows enhanced apoptosis due to reduced autophagic flux [75]. Other groups have shown similar dependence of CD4^+^ and CD8^+^ T cells on autophagy protein Atg5 and Atg7 for their development, proliferation and survival [76].

Impaired primary antibody responses to antigen immunization and defective plasma cell differentiation have been demonstrated in autophagy deficient B cells [77]. In contrast, other studies found no defects in primary antibody responses from autophagy-deficient B cells following antigen immunization [78] as well as no change in the number of mature B cells [77, 79]. The dissimilarities in these findings may be due to differences in immunization regimes, antigen and adjuvants used [57]. Secondary antibody response and survival of memory B cells, is found to be largely dependent on the process of autophagy [78, 80]. These results are comparable to memory CD8^+^ T cell responses during viral infections where autophagy is also involved in memory cell formation during the late stages [57, 81]. The number of B cells in the intestinal lamina propria and Peyer’s patches in *Atg5*^*ΔCD19*^ (Atg5 deficient B cells) mice are significantly decreased compared to the wild type. However, B cell numbers in the spleen and bone marrow are similar when compared to wild type and *Atg5*^*ΔCD19*^ mice. This observation leads us to speculate that the role of autophagy in maintaining intestinal B cell physiology is more important compared to that in the peripheral B cells, although further investigation is needed [57, 77].

### Autophagy and gut microbiota

**Fig. 2:**
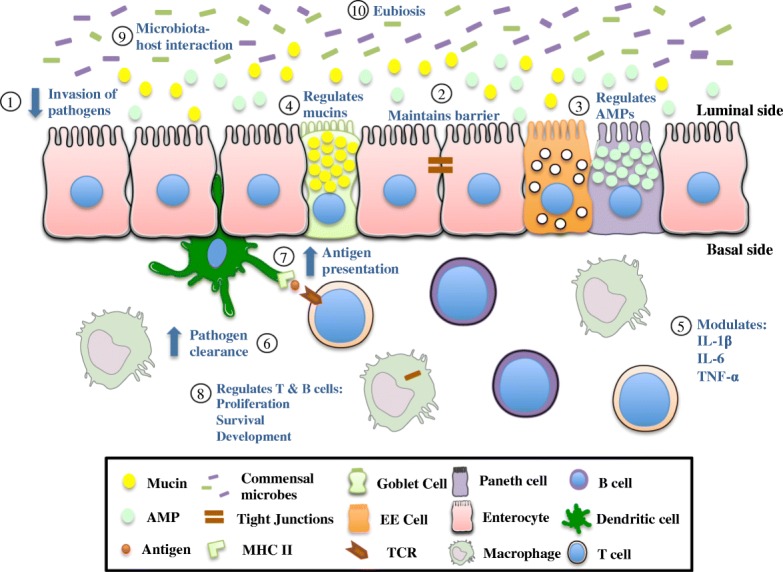
Schematic representation of the role of normal autophagy in maintaining intestinal homeostasis. The intestinal mucosa includes several types of epithelial and immune cells. This schematic illustrates the importance and function of normal autophagy in the intestinal mucosa. Autophagy is critical in preventing the invasion and dissemination of pathogens (1) and maintaining barrier integrity (2). Autophagy regulates the secretion of AMPs from Paneth cells (3), the secretion of mucins from goblet cells (4) and the differentiation of EE cells in intestinal epithelial lining. Autophagy is involved in different immunological functions such as cytokine secretion (5), pathogen clearance by macrophages (6), antigen presentation by DCs (7), effector and memory T cell development (8), and secondary antibody response. The process of autophagy can regulate the interaction between the host and the gut microbiota (9). Autophagy also helps to maintain the balance between harmful and beneficial bacteria of the gut (10)

More than 100 trillion bacteria inhabit the lower gastrointestinal tract of humans. Thus, the number of microbes in the human body exceeds the number of host cells by about 10 times. The gut microbiota is influenced by factors such as age, gender, diet and immune status [82] and the interaction between the host and these microbes is largely symbiotic. The symbiotic bacteria provide the human host with nutrients, facilitate digestion and prevent colonization of opportunistic pathogens in the gut [83]. However, under altered conditions, even the normal microbiota may induce inflammation. The gut microbiota is implicated in various gastrointestinal disorders including IBD. Clinical and animal studies have suggested that gut bacteria trigger and perpetuate chronic colitis [84]. Very recently we also demonstrated that 5-HT-microbiota axis plays an important role in gut inflammation. Higher gut mucosal 5-HT levels select for a more colitogenic microbiota, resulting in increased severity of colitis [85]. The mTOR signaling pathway has a key role in microbiota associated immune regulation and intestinal disease development [82]. Noureldein and colleagues [82] have eloquently summarized the association between microbiota and mTOR signaling. The intestinal microbiota and its metabolites regulate various physiological functions of the host and maintain homeostasis through the mTOR pathway, which controls many cellular processes, autophagy being one of them. Increased activation of AMPK in the liver, muscle and colon in germ-free mice provide evidence that gut microbiota regulates activation of AMPK in the host [82]. Both autophagy and gut microbiota are important factors in the development of CD. Recently, the composition and diversity of gut microbiota has been reported to be very different in IEC specific *Atg5* conditional KO mice [86]. Beneficial anti-inflammatory bacteria such as the mucin degrading *Akkermansia muciniphila* and members of the Lachnospiraceae family are decreased in the *Atg5* IEC specific KO mice model. On the other hand, pro-inflammatory bacteria such as *Candidatus athromitus* and potential pathogens of the Pasteurellaceae family are increased in the same *Atg5* IEC specific KO mice. To further elucidate the connection between autophagy and microbiota, it has been found that protective factors like vitamin D_3_ induce autophagy in colitis model and promotes a healthier microbiota composition [87]. Vitamin D receptor (*VDR*) IEC specific KO mice are reported to have reduced amount of Atg16L1 at both the transcriptional and protein level compared to their wild type counterparts. In addition, it has been shown that Atg16L1 is required for the proper functioning of AMP producing Paneth cells that regulate the composition and diversity of gut microbiota [32]. *VDR* IEC specific KO mice with reduced Atg16L1 and defective Paneth cell have increased bacterial loads and a shift of microbial species [88]. Harmful bacteria such as *E. coli* and *Bacteroides* are increased whereas beneficial butyrate-producing bacteria are decreased in the *VDR* IEC specific KO mice. *VDR* IEC specific KO mice also developed more severe DSS induced colitis compared to the wild type mice, implying that microbial dysbiosis may increase the susceptibility of colonic mucosa to DSS induced colitis. The administration of the short chain fatty acid, butyrate to *IL-10* KO mice increased mRNA levels of Atg16L1, suppressed IL-6 production and restored Paneth cell numbers. These findings indicate that the bidirectional relationship between the host and microbiota is regulated by a functioning autophagy system in the host intestinal epithelium [88]. So far, we discussed the effects of impaired autophagy components on the gut microbiota. However, as mentioned earlier this relationship is bidirectional, i.e. autophagy can influence gut microbiota and gut microbiota can also influence autophagy in the host. It has been reported that microbiota derived metabolites regulate intestinal inflammation by acting through the autophagy pathway. Trimethylamine N-oxide, a gut microbiota derived metabolite increased IEC nucleotide-binding domain, leucine-rich-containing family, pyrin domain-containing-3 (NLRP3) inflammasome activity by inhibiting Atg16L1, LC3-II and p62 expression [89]. *Bifidobacteria,* a probiotic strain in the human gut microbiota initiates autophagy, probably through the Atg12–5-16 L1 pathway in IEC culture model [90] further highlighting the importance of interaction between microbiota and autophagy in IEC. Nevertheless, functional consequences of such interaction between gut bacteria and autophagy in the IEC remain to be determined. The homeostatic functions of autophagy in intestinal mucosa have been summarized in Fig. 2.

### Autophagy and gut inflammation

Genome-wide association studies (GWAS) identified an increased risk of developing CD in individuals with nonsynonymous single nucleotide polymorphism (SNP) at the *Atg16L1* locus [9]. The SNP caused a threonine to alanine substitution at position 300 (T300A) of the protein Atg16L1 [9]. Nearly a decade later after this discovery, Murthy and colleagues [91] provided evidence of increased caspase-3 mediated degradation of T300A variant of Atg16L1. The caspase cleavage site was identified as the site immediately preceding the T300A polymorphism site [91]. There is a 50% reduction in Atg16L1 protein levels in glucose starved macrophages isolated from *T316A* knock in (T300A variant in mice) mice with resultant decrease in bacterial degradation by autophagy and increased expression of the pro-inflammatory cytokine IL-1β. This resulted in defective stress activated autophagy and xenophagy, thus establishing a state of chronic inflammation that predisposes individuals to CD [91]. Another autophagy gene, immunity related GTPase family M (*IRGM*), has also been linked with CD [92]. *IRGM* polymorphisms are responsible for dysfunctional autophagy which perpetuates the inflammatory process in IBD [93]. Further supporting this link is the finding that Atg16L1 and IRGM protein expression is reduced in the intestine of CD patients [94]. Autophagy has been linked in the pathogenesis of CD through nucleotide-binding oligomerization domain-containing protein 2 (*NOD2*) gene, a genetic risk factor for CD development. Activation of NOD2 by bacterial products like muramyl dipeptide recruits Atg16L1 to the isolation membrane and stimulates the formation of autophagosomes [68, 95]. Stimulation of NOD2 in human primary immune cells results in increased pro-inflammatory cytokine response when autophagy is inhibited [96]. In CD patients, *NOD2* and *Atg16L1* polymorphism demonstrate similar impairment of NOD2 dependent autophagy, further highlighting the importance of autophagy in CD [68, 95]. *Lrrk2*, another CD susceptibility gene, has been linked with xenophagy and Paneth cell autophagy. Deletion of *Lrrk2* results in impaired Paneth cell autophagy, which contributes to the onset of IBD [97, 98]. It is clear from the findings of GWAS that dysfunctions in autophagy genes contribute to the development of intestinal inflammation.

Several mechanisms have been proposed to explain the link between autophagy and gut inflammation. Ravindran and colleagues [99] reported enhanced intestinal inflammasome activation, increased production of IL-1β, increased Th17 response and reactive oxygen species formation in a *GCN2* (general controlled non-repressed kinase) KO mouse model. They further found that reduced autophagy in IECs and APCs in *GCN2* KO mice is responsible for intestinal inflammation [99]. The link between autophagy and inflammasome has been studied in mice models with *Atg16L1* and *Atg7* deficient macrophages [63]. Treatment of *Atg16L1* and *Atg7* deficient macrophages with LPS, a TLR-4 ligand, results in markedly elevated levels of IL-1β. However, stimulation of TLR-2 or TLR-5 does not have the same effect. The levels of IL-1β mRNA and pro-IL-1β protein are similar in the *Atg16L1* deficient and wild type macrophages. This showed that autophagy regulates IL-1β production in the post-translational stage. It has been found that LPS stimulation mediates the cleavage of caspase-1 through activation of inflammasome, which results in the release of IL-1β from pro-IL-1β. These evidence suggest that in the absence of autophagy there is excessive cytokine production through inflammasome activation [63, 100]. Autophagy is thought to regulate inflammasome activity mainly through two mechanisms; (i) it causes defects in mitophagy resulting in increased reactive oxygen species production which is linked with inflammasome hyperactivity, and (ii) it negatively regulates pro-IL-1β and other components of inflammasome pathway [101]. Autophagy prevents an exaggerated pro-inflammatory response in the gut by other mechanisms such as by influencing the immune response to commensal bacteria, by regulating mucus secretion, by preventing accumulation of aggregates and by regulating miRNA-silencing pathways [27, 99, 102].

The in vivo role of autophagy proteins in gut inflammation has been examined in experimental models of colitis. Different autophagy deficient mice models such as *Atg16L1* deficient chimeric mice, myeloid *Atg16L1* deficient mice, colonic epithelial cell-specific *Atg7* conditional KO mice, and *Atg4B* null mice all exhibit exacerbated colitis induced by DSS [34, 63, 64, 103]. None of the autophagy deficient mice models develop spontaneous inflammation. However, severe spontaneous transmural ileitis was reported when both unfolded protein response and autophagy function are impaired in IECs [104]. On the other hand, the severity of spontaneous enterocolitis in *IL-10* KO mice decreased with increased autophagy [105, 106]. Pharmacological activation of mucosal autophagy using rapamycin or other mTOR-inhibitors results in reduction of intestinal inflammation in experimental models of colitis and in severe refractory CD. All these studies suggest that enhancing autophagy in the gut plays a protective role against experimental colitis [107–109].

**Fig. 3:**
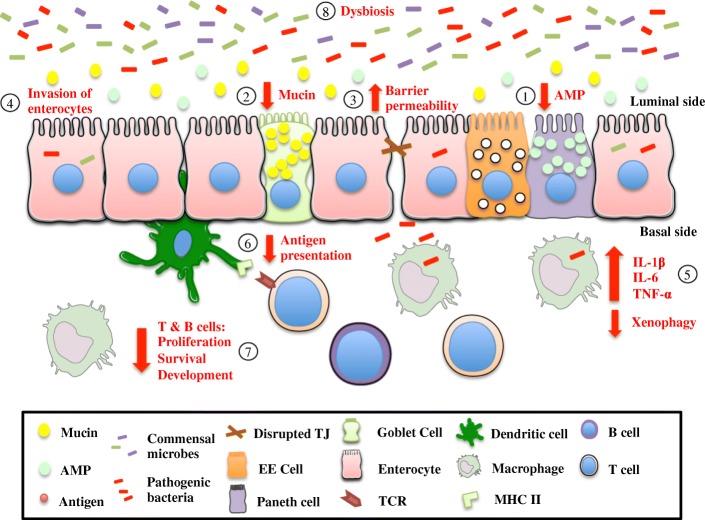
Schematic representation of effect of impaired autophagy in intestinal mucosa. Defects in the autophagy system result in breakdown of mucosal homeostasis of the intestine. This diagram shows the state of the intestinal epithelium, mucosal immune system and microbiota in the context of impaired autophagy. Defective autophagy results in decreased AMP (1) and mucin (2) production, increased barrier permeability (3), increased bacterial invasion and dissemination (4), impaired cytokine production (5), reduced antigen presentation (6) and reduced T and B cell survival and development (7). Disrupted autophagy can alter the composition of bacteria within the gut, trending towards increased levels of pro-inflammatory species and decreased levels of anti-inflammatory species, thus, resulting in dysbiosis (8). Autophagy is required for the proper function of the gut and prevention of an exaggerated pro-inflammatory response

Until now the mechanism by which impaired autophagy promotes a pro-inflammatory condition has mostly been explained in the context of the immune system with focus on the inflammasome. However, Grizotte-Lake and colleagues [110] recently summarized the role of autophagy in IECs in the pathogenesis of IBD [110–113]. Autophagy deficient IECs are found to be more susceptible to TNF-α induced damage and cell death. Using either murine norovirus or *Helicobacter hepaticus* or *T. gondii* infection mice models, it has been demonstrated that when autophagy is impaired in the intestinal epithelium, increased amounts of TNF-α mediates cell death. In the murine norovirus infection model, IEC specific *Atg16L1* KO mice are more susceptible to DSS induced colitis [112]. Atg16L1 protein in the IECs prevents loss of Paneth cells and TNF-α mediated necroptosis (programmed necrosis) in the intestinal epithelium of virally triggered IBD mouse model. In the same infection model, TNF-α blockage inhibits necroptosis in the IECs. Opportunistic pathogen, *H. hepaticus* triggered chronic colitis mice are recorded to have analogous findings of increased IEC apoptosis (programmed cell death) following conditional deletion of *Atg16L1* in the IECs [113]. This exaggerated IEC apoptosis is reported to be due to increased susceptibility to TNF-α and, as expected, TNF-α inhibition reduces the colitis severity in *Atg16L1* conditionally KO mice. Lastly, a *T. gondii* infection model has been employed to reach the same conclusion of increased sensitivity to TNF-α induced inflammation in autophagy deficient Paneth cells [111]. During *T. gondii* infection in a Paneth cell *Atg5* KO mouse model, there is heightened intestinal inflammation due to increased susceptibility to pro-inflammatory cytokine TNF-α. Therefore, *Atg5* expression in Paneth cells plays a vital role in protecting against acute gastrointestinal infection. Even though in healthy state IECs can function normally without autophagy, it is in the event of an infection that autophagy in the intestinal epithelium is required to prevent cytokine induced cell death and uncontrolled inflammation. The association between TNF-α and autophagy in these three models helps to elucidate the mechanism behind anti-TNF-α therapy in IBD. It will be interesting to determine whether IBD patients with *Atg16L1* polymorphism are more responsive to anti-TNF-α therapy. More importantly, these findings may help optimize treatment protocols and maximize treatment success rates for IBD patients. Neonatal necrotizing enterocolitis (NEC) rat small intestine and NEC cell line showed increased expression of TNF-α and autophagy levels compared to the controls. TNF-α increased autophagy levels in NEC cells as evidenced by increased beclin-1 and LC3-II/I ratio. Additionally, inhibition of autophagy by wortmannin or LY294002 prevents TNF-α induced inhibition of proliferation and the promotion of apoptosis. Results also indicate that inhibition of ERK1/2 pathway significantly reduces TNF-α induced autophagy [114]. Therefore, it can be suggested that in both colitis and NEC models, TNF-α regulates apoptosis in intestinal epithelial cells via the process of autophagy. The role of autophagy in inflamed intestinal mucosa has been illustrated in Fig. 3.

## Discussion and conclusion

Autophagy, a physiological process, is crucial in maintaining intestinal physiology and in controlling mucosal inflammation. Since the 1960s, the mechanisms and pathways of autophagy have been studied extensively and are still being explored today [5]. The discovery of Atg5/Atg7 independent pathway suggests that distinct autophagy pathways and molecules might be specific for different types of cells. This highly conserved process has tremendous influence on the normal functioning of the various cells of the intestine including epithelial and immune cells. Autophagy is maintained in these cell types for its support in basic functions such as maintaining pools of amino acid, as well as, removal of defective organelles [5]. Apart from these fundamental roles, autophagy regulates special functions of the intestinal mucosa. In the epithelial lining, autophagy is critical in preventing the invasion and dissemination of pathogens, maintaining barrier integrity and preserving intestinal homeostasis.

The findings of the GWAS on CD opened new avenues of research in gut autophagy in relation to pathology and pathogenesis of gut inflammation. Evidence of autophagy regulating secretion of AMP from Paneth cells, secretion of mucins from goblet cells and differentiation of EE cells emphasizes the diverse role of autophagy in the intestinal epithelial layer. Autophagy is also involved in different immunological functions such as cytokine secretion, pathogen clearance by macrophages, antigen presentation by DCs, effector and memory T cell development and secondary antibody response [57]. The gut microbiota and autophagy have also been linked and this relationship appears to be bidirectional. It is quite clear that dysregulation of autophagy is associated with the pathophysiology of various intestinal diseases, notably IBD. GWAS have revealed a link of autophagy genes with CD, marking the beginning of a new era in intestinal inflammation research. It is now recognized that autophagy in the gut is protective against experimental colitis and that patients with CD have dysfunctional autophagy proteins suggesting that autophagy is required for the proper function of the gut and prevention of exaggerated pro-inflammatory response.

In recent years, significant progress has been made in understanding the mechanisms of autophagy and the role it plays in regulation of various key cells involved in intestinal inflammation including IECs and immune cells. Autophagy modulators such as vitamin D and rapamycin are currently being studied as potential therapeutic agents in intestinal inflammation [11]. The effectiveness of rapamycin has already been documented in a case of severe refractory CD [109]. Even though our understanding of autophagy has improved, this topic needs to be explored further before these pathways can be manipulated to treat human diseases such as IBD. As basal level of autophagy occurs within all cells, autophagy regulating drugs need further investigation as substances affecting this process may have diverse and global implications. In addition, it will be particularly important to gain further knowledge on the interactions between autophagy and various mediators like cytokines, neurotransmitters and hormones, which are upregulated during intestinal inflammation. Identification of novel regulators of autophagy in gut inflammation may trigger development of new therapeutic agents for intestinal inflammatory disorders such as IBD, and other GI disorders that display dysregulated autophagy.

## Abbreviations

AMPK: Adenosine monophosphate-activated protein kinase
AMPs: Anti-microbial peptides
Atg: Autophagy-related protein
CD: Crohn’s disease
CgA +: Chromogranin A positive.
CMA: Chaperone-mediated autophagy
DC: Dendritic cells
DSS: Dextran sulphate sodium
EE: Enteroendocrine
ER: Endoplasmic reticulum
FIP200: Focal adhesion kinase family interacting protein of 200 kDa
GCN2: General controlled non-repressed kinase
GWAS: Genome-wide association studies
HIF-1: Hypoxia inducible factor 1
HSC70: Heat shock cognate 70 kDa protein
IBD: Inflammatory bowel disease
IEC: Intestinal epithelial cell
IRGM: Immunity related GTPase family M
LAMP2A: Lysosome-associated membrane protein 2A
LC3: Microtubule-associated protein 1A/1B-light chain 3
LPS: Lipopolysaccharide
MHC II: Major histocompatibility complex II
mTOR: Mammalian target of rapamycin
NADPH: Nicotinamide adenine dinucleotide phosphate hydrogen
NEC: Necrotizing enterocolitis
NLRP3: Nucleotide-binding domain, leucine-rich-containing family, pyrin domain-containing-3
NLRP6: Nucleotide-binding oligomerization domain protein-like receptors protein 6
NOD2: Nucleotide-binding oligomerization domain-containing protein 2
PAS: Phagophore assembly site
PE: Phosphatidylethanolamine
PI3P: Phosphatidylinositol-3-phosphate
PI3K: Phosphatidylinositol-3-kinase
SNP: Single nucleotide polymorphism
TCR: T cell receptor
TJ: Tight junction
TLR: Toll-like receptor
UC: Ulcerative colitis
ULK1: Unc-51 like autophagy activating kinase
VAMP8: Vesicle-associated membrane protein 8
VDR: Vitamin D receptor
Vps: Vacuolar protein sorting
